# Allee effects and the Allee-effect zone in northwest Atlantic cod

**DOI:** 10.1098/rsbl.2021.0439

**Published:** 2022-02-02

**Authors:** Tommi Perälä, Jeffrey A. Hutchings, Anna Kuparinen

**Affiliations:** ^1^ Department of Biological and Environmental Science, University of Jyväskylä, PO Box 35, Jyväskylä 40014, Finland; ^2^ Department of Biology, Dalhousie University, 1355 Oxford Street, Halifax, Nova Scotia, Canada B3H 4R2; ^3^ Institute of Marine Research, Flødevigen Marine Research Station, N-4817 His, Norway; ^4^ Department of Natural Sciences, University of Agder, N-4604 Kristiansand, Norway

**Keywords:** *Gadus morhua*, compensation, depensation, low-abundance dynamics, marine conservation, stock–recruitment relationship

## Abstract

According to the theory of compensatory dynamics, depleted populations should recover when the threat responsible for their decline is removed because *per capita* population growth is assumed to be highest when populations are at their smallest viable sizes. Yet, many seriously depleted fish populations have failed to recover despite threat mitigation. Atlantic cod (*Gadus morhua*) stocks off Newfoundland, despite 30 years of dramatically reduced fishing mortality and numerous fishery closures, have not recovered, suggesting that drivers other than fishing can regulate the growth of collapsed fish populations, inhibiting or preventing their recovery. Here, using Bayesian inference, we show strong evidence of Allee effects in a south Newfoundland cod population, based on data on recruitment and spawning stock biomass. We infer the Allee-effect threshold, below which recovery is impaired. We demonstrate the necessity of data at low population sizes to make inferences about the nature of low-abundance dynamics. Our work indicates that Allee effects are not negligible in commercially exploited fish populations, as commonly projected, and that they represent an inhibitory force that can effectively prevent recovery from overfishing. Our findings contrast with prevailing fisheries management practices that assume compensatory dynamics at low abundances with potential to seriously overestimate the recovery potential of collapsed populations.

## 1. Introduction

Vulnerability assessments and conservation strategies are commonly based on the premise that the primary factor preventing the recovery of a depleted species is the threat responsible for its decline. Remove or mitigate the threat and recovery will follow. However, while threat mitigation is clearly necessary to halt species or population decline, threat abatement in and of itself is not always sufficient to result in recovery [1]. Independently of the threats responsible for decline, the magnitude of depletion itself can impair or increase the uncertainty of recovery if populations fall below a threshold density or population size below which realized *per capita* growth rate (*r*) declines with increasingly smaller population size. This threshold is termed the Allee-effect threshold [2] and the resultant pattern of positive density dependence (*r* increasing with population size) is termed an Allee effect [3,4].

Although Allee effects have been acknowledged as a potential inhibitor of species recovery [2,5,6], their explicit quantitative consideration has been hampered by an empirical paucity of data on which the Allee-effect threshold can be estimated. Further complicating the identification of the threshold is the question of whether the manifestation of Allee effects represents a sharp or gradual transition from negative to positive density-dependent dynamics.

Allee effects are empirically challenging to study [7–9]. It can be extremely difficult to disentangle the effects of reduced population size or density *per se* on recovery from the effects of other inhibiting factors, such as habitat alteration or destruction. However, unlike most species, many marine fish populations have suffered declines that can primarily be attributed to a single over-arching threat: overfishing. For this reason, and the ready availability of lengthy time series of population sizes, marine fishes represent an opportune group of species in which to study Allee effects, referred to in the fisheries literature as ‘depensation’.

The relationship between the number of offspring produced (recruitment, *R*) and the size of the parental population (spawning stock biomass, *S*) is commonly used to detect Allee effects in marine fishes [10,11]. Standard stock–recruitment (*S–R*) models assume compensatory dynamics, i.e. the slope of the *S–R* relationship increases (becomes steeper) as *S* becomes increasingly small. The steepness of the relationship is used as a measure of the strength of compensatory population dynamics: the steeper the slope, the greater the recovery potential and the smaller the spawning stock biomass at which the maximum sustainable yield can be obtained [12]. The traditional compensatory *S–R* models do not, however, allow enough flexibility to explain Allee effects in the data. Thus, more flexible models allowing depensatory dynamics are used to reflect stock–recruitment relationships for which *R*/*S* (a proxy for *r*, related to *per capita* production of offspring) declines, rather than increases, as *S* approaches zero [13–15].

Atlantic cod (*Gadus morhua*) is an iconic commercially and ecologically important species found throughout the North Atlantic. Following several collapses in the late 1980s and early 1990s [16,17], all eight Canadian cod stocks were subjected to fishery closures, most of which remain in effect today. One of these stocks, however, was closed for only 3 years (1994–1996), after which Canadian catches rapidly increased (under quota management) to the pre-moratorium levels of the 1980s. This St Pierre Bank stock (Northwest Atlantic Fisheries Organization Subdivision 3Ps), positioned along much of the south coast of Newfoundland, was certified as sustainable by the Marine Stewardship Council in March 2016; the certification was suspended in May 2017. Population size estimates of 3Ps cod have long been considered problematic because of spatial differences in stock components and their seasonal movements. However, since 2020, a new, integrated state-space model has been applied to commercial catch data (1959–), time-varying estimates of natural mortality, and abundance indices from multiple fisheries-independent surveys, to produce more reliable estimates of population abundance [18,19].

Here, we apply four *S–R* models (table 1) to (i) test for evidence of Allee effects in St Pierre Bank Atlantic cod, (ii) infer the Allee-effect threshold, and (iii) determine the time frame over which the switch from negative to positive density-dependent dynamics occurs.

**Table 1.** Stock–recruitment models. Names, abbreviations, expected number of recruits $E(R_{t}|S_{t},\theta )$ and unknown parameters *θ* and their prior distributions. The variables appearing in the table are the number of recruits (*R_t_*), spawning stock biomass (*S_t_*), the asymptotic maximum number of recruits (*R*_∞_), the spawning stock at which the number of recruits is ½*R*_∞_ in BH and SBH models (*S*_50_), the maximum number of recruits (*k*), the spawning stock at which the number of recruits is *k* in RI and SL models (*S_k_*), the depensation parameter *c*, the upper bounds for the supports of the parameters related to the maximum number of recruits (R*) and the corresponding parameters for the spawning stock biomass (S*), respectively.

name	abbreviation	*E* (*R_t_*|*S_t,_θ*)	*θ*
Beverton–Holt	BH	$\frac{R_{\infty }}{1+(S_{50}/S_{t})}$	$R_{\infty }∼U(0,R^{*})$
			$S_{50}∼U(0,S^{*})$
Ricker	RI	$k\frac{S_{t}}{S_{k}}e^{1-(S_{t}/S_{k})}$	$k∼U(0,R^{*})$
			$S_{k}∼U(0,S^{*})$
Sigmoidal Beverton–Holt	SBH	$\frac{R_{\infty }}{1+{\left(S_{50}/S_{t}\right)}^{c}}$	$R_{\infty }∼U(0,R^{*})$
			$S_{50}∼U(0,S^{*})$
			c∼π(u,q)
Saila–Lorda	SL	$k{\left(\frac{S_{t}}{S_{k}}\right)}^{c}\;e^{c(1-(S_{t}/S_{k}))}$	$k∼U(0,R^{*})$
			$S_{k}∼U(0,S^{*})$
			c∼π(u,q)

## 2. Material and methods

### (a) Stock–recruitment data

The data on *S* (electronic supplementary material, figure S1) and *R* (electronic supplementary material, figure S2) of Atlantic cod in NAFO subdivision 3Ps management area were extracted from the Canadian Science Advisory Secretariat Newfoundland and Labrador region science advisory report 2021/031 [19] (see also estimates of fishing mortality *F*; electronic supplementary material, figure S3). The recruitment and spawning stock biomasses are estimated from their new population model. Data cover the years 1959 to 2017.

### (b) Stock–recruitment models

We examine the *S–R* data using four different models, namely, Beverton–Holt [20], Ricker [21], Sigmoidal Beverton–Holt [14] and Saila–Lorda [22,23]. For each model, we model the number of recruits *R_t_* given the spawning stock biomass *S_t_*, the unknown parameters of the *S–R* models *θ* and the unknown scale parameter *σ*^2^ using a lognormal distribution

$$R_{t}|S_{t},\theta ,\sigma^{2}∼\mathrm{Lognormal}\;(\mu_{t},\sigma^{2}),$$

where the location parameter *µ_t_* is expressed in terms of the expected number of recruits $E(R_{t}|S_{t},\theta )$

$$\mu_{t}=\log(E(R_{t}|S_{t},\theta ))-\frac{1}{2}\sigma^{2}.$$

The *S–R* models, their abbreviations, formulae for the expected number of recruits, and the unknown parameters and their prior distributions are shown in table 1. The supports of the *S–R* model parameters have upper bounds R* and S*, and their effect on the results was studied. The choice of the prior distributions and parameter supports are further described in the electronic supplementary material.

### (c) Allee-effect zone and threshold

For the depensatory versions of SL and SBH (i.e. *c* > 1), we define the Allee-effect threshold, *S*_0_, as the value of *S* where the *S–R* function changes from convex to concave. We can analytically solve for *S*_0_ for both models. For SL, the Allee-effect threshold is

$$S_{0}=\left(1-\frac{1}{\sqrt{c}}\right)S_{k},$$

and for SBH

$$S_{0}=\frac{S_{50}}{{\left(\left(c\;+\;1)/(c\;-\;1\right)\right)}^{1/c}},$$

when the depensation parameter *c* ≤ 1, the Allee-effect threshold is not defined. When the other model parameters are estimated, it is straightforward to estimate the value of the Allee-effect threshold. The Allee-effect zone is then defined as the convex part of the S–R function nearest to the origin, i.e. the interval [0, *S*_0_].

### (d) Model fitting and comparison

We conducted the posterior inference sequentially, starting with one data point in year 1959 and adding each consecutive year one by one to demonstrate how the evidence of low-abundance dynamics is accumulated and the uncertainty about the model parameters and predictions is reduced as time goes by and new data are observed. We compare the model fits using the widely applicable information criterion (WAIC) [24,25], which scores the models based on their pointwise out-of-sample prediction accuracy, and we calculate the posterior probability of depensation for each year and each (depensatory) model by examining the posterior probability distributions of the depensation parameter.

### (e) Implementation and statistical inference

The models were implemented, and the posterior inference carried out using the probabilistic programming language Stan [26]. The burn-in period was 10 000 iterations, after which the next 100 000 samples were recorded. The convergence of the models was checked visually.

## 3. Results

Sequential inspection of the model parameter posterior probability distributions and WAIC brings valued temporal resolution to the analysis. First, according to the WAIC, all four models explain the data almost equally well until the mid-1970s, after which the performance of the traditional compensatory models (RI and BH) drastically decreased (electronic supplementary material, figure S4a). Both depensatory models (SL and SBH) perform well throughout the study. The depensatory models also outperform a ‘null’ model where recruits-per-spawner is assumed to be constant (see electronic supplementary material, figures S5 and S6).

The failure of the compensatory models to adequately explain the data is our first piece of evidence for the existence of an Allee effect in St Pierre Bank Atlantic cod. The second piece of evidence is seen in the posterior probability of depensation (electronic supplementary material, figure S4b). For the SL and SBH models, the probability of depensation rises above 0.9 after 1976, reaches 1 soon thereafter, and remains unchanged until the end of the study period. These results are not sensitive to the choice of R* and S* (electronic supplementary material, figure S7).

**Figure 1.**
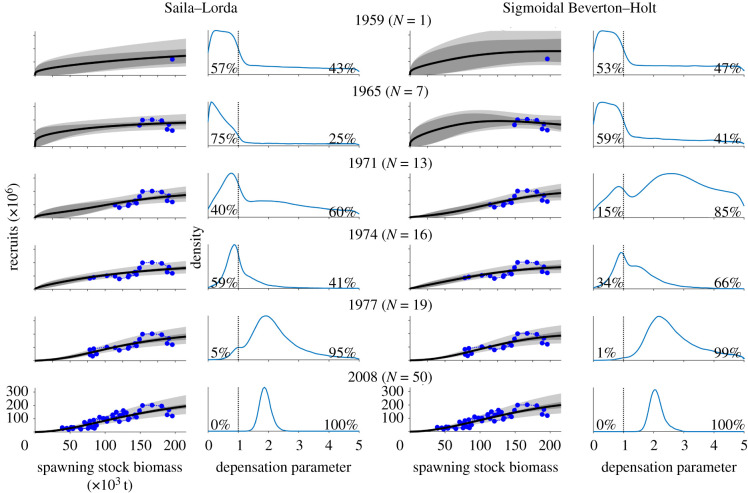
Snapshots of the model-fitting process on selected years. The two leftmost columns show the snapshots for SL and the two rightmost columns for SBH. The column on the left of each pair of columns shows the model fit to the data accumulated by a given year. Blue dots show the stock–recruitment data, black lines show the means of the posterior predictive distribution of recruits as a function of *S*, the dark and light grey areas show the 68% central probability intervals of the expected values of the models and the posterior predictive distributions, respectively. The column on the right of each pair of columns shows the posterior probability density functions of the depensation parameter *c* (*c* > 1 indicates depensation). The probability of compensation/depensation is shown at the bottom left/right corner of each graph.

Closer inspection of the model parameter posterior probability distributions and the model fits in terms of the posterior predictive distributions reveals how in the beginning of the study period, the evidence of depensation ranges from neutral to weak (figure 1, years 1959 and 1965). As data at low spawning stock biomass levels become increasingly available, the evidence for depensation quickly becomes very strong (figure 1, year 1977 onwards). Figure 1 also shows how the uncertainty about the low-abundance dynamics is very high when there are no observations at low levels of spawning stock biomass (years 1959 and 1965) and how the uncertainty decreases as observations at low spawning stock biomasses become available (figure 1, year 1977 onwards). The reduced uncertainty about the nature of the dynamics is also reflected in the narrowing of the posterior distribution of *c*. Similar behaviour was also observed for decreased R* and S* (electronic supplementary material, figure S8).

**Figure 2.**
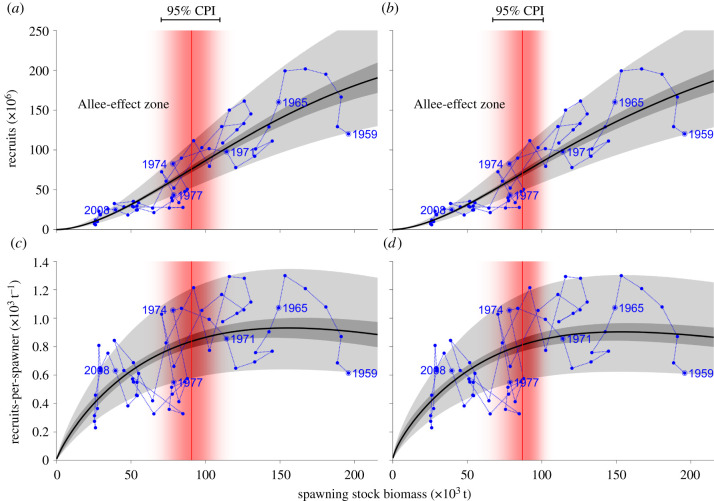
Model fits and the Allee-effect thresholds. On the top row (*a*,*b*) the data on *S* and *R* are shown in blue, whereas on the bottom row (*c*,*d*) the vertical axis shows *R*/*S*. The snapshot years of figure 1 are labelled and shown with dots with circles around them. The black line shows the mean of the posterior predictive distribution of *R* or *R*/*S* as a function of *S*. The dark and light grey areas show the 68% central probability intervals (CPI) of the expected values of the model and the posterior predictive distributions, respectively. The red area shows the posterior distribution of *S*_0_. The area to the left of the Allee-effect threshold is called the Allee-effect zone; it is in this zone that the stock–recruitment dynamics becomes depensatory. The left column (*a*,*c*) shows output from SL and the right column (*b*,*d*) from SBH.

With 100% posterior probability of *c* > 1, it is possible to calculate the Allee-effect threshold *S*_0_ below which positive density dependence begins to manifest. The 95% central probability intervals of the Allee-effect thresholds identify a narrow range of *S* within which the shift from negative to positive dependence occurs (figure 2). The thresholds for the SL and SBH models are 90 400 t (70 300–109 600 t) and 86 200 t (67 100–101 000 t), respectively. The decline in *R*/*S* begins at *S* larger than the Allee-effect threshold, revealing a broad range of spawning stock biomasses at which negative density dependence is weak, suggestive of a broad Allee transition region [10]. Halving R* and S* decreased the uncertainty about *S*_0_ while also decreasing its expected value, yielding Allee-effect thresholds for the SL and SBH models of 65 400 t (56 800–73 200 t) and 61 600 t (52 600–68 800 t), respectively (electronic supplementary material, figure S9).

## 4. Discussion

Our work provides the first evidence of demographic Allee effects in Atlantic cod, as reflected by a decline in per capita production of offspring (*R*/*S*) with reductions in the size of the parental population. The percentage of maximum observed spawning stock biomass at which the Allee-effect threshold occurs in St Pierre Bank cod is 46.3% (SL) or 44.3% (SBH) of *S*_max_.

Our work has practical implications for the management and attainment of sustainable fisheries. Under the 1995 UN Fish Stocks Agreement [27], sustainable fisheries management is guided by the setting of reference points that delineate ranges of population size that should be avoided, demarcated by a biomass limit reference point or *B*_lim_. Interestingly, the *B*_lim_ = 66 000 t set by Fisheries and Oceans Canada (DFO) fisheries scientists for the St Pierre Bank cod population [18] is very close to the 2.5% quantile Allee-effect thresholds (70 300 t and 67 100 t) identified in the present study. Although the DFO-based *B*_lim_ did not explicitly account for Allee effects, their analysis allowed for the establishment of *B*_lim_ as the level of *S* above which *R* increases with *S*. Given that the intent of *B*_lim_ is to identify a population size below which irreparable harm to population viability has heightened probability of occurrence, the use of depensatory *S–R* models to identify *B*_lim_ has considerable merit.

Our methods can be used in conjunction with other methods to determine *B*_lim_ with the additional precautionary benefit that it can identify population sizes greater than *B*_lim_ at which the probability of Allee effects being realized is small. Analytically, this is reflected by an increase in the posterior probability that the depensation parameter *c* exceeds 1. A weakening of negative density dependence, notwithstanding the prospects of positive density dependence, can be sufficient to impair recovery.

Our demonstration of demographic Allee effects in St Pierre Bank cod lends credence to previous assertions that marine fish populations can experience positive density-dependent population dynamics [2,11,28]. Addressing methodological issues associated with previous analyses [13], a Bayesian statistical analysis documented depensation in 4 of 9 Atlantic herring (*Clupea harengus*) populations [15]. Evidence of emergent Allee effects, generated by altered predator–prey interactions with declining prey abundance (so-called ‘predator pit’), has been reported for the Southern Gulf of St Lawrence cod population in the northwest Atlantic [29]. However, for Southern Gulf cod, *R*/*S* does not decline with decreasing *S*, but another proxy for *r* (natural mortality, *M*) does.

Similar models have been applied previously to the study of Allee effects in nine Atlantic herring stocks [15]. Here, we develop the methodology further by introducing the analytical formulation of the Allee-effect threshold derived from the stock–recruitment models and infer its posterior distribution. Also, unlike the previous study, we demonstrate sequentially in time how the evidence of depensation is accumulated. Furthermore, we introduce an information criterion-based model comparison as further evidence for depensation using the widely applicable information criteria.

As empirical evidence on Allee effects in marine fishes accumulates, the default assumption made in fisheries stock assessments regarding compensatory dynamics is outdated and inconsistent with a precautionary approach to sustainable fisheries management. Given that Allee effects can substantially slow down or even prevent recovery from overfishing, sustainable fisheries management should, firstly, account for the possibility that Allee effects exist and, secondly, maintain populations well above the abundances below which those start to manifest.

## Data accessibility

The data and codes are availble in Dryad: http://dx.doi.org/10.5061/dryad.r4xgxd2dg [30].
